# Heat shock factor 1 over-expression protects against exposure of hydrophobic residues on mutant SOD1 and early mortality in a mouse model of amyotrophic lateral sclerosis

**DOI:** 10.1186/1750-1326-8-43

**Published:** 2013-11-21

**Authors:** Pei-Yi Lin, Sharotka M Simon, Won Kyun Koh, Oluwarotimi Folorunso, C Samuel Umbaugh, Anson Pierce

**Affiliations:** 1 Department of Biochemistry & Molecular Biology, University of Texas Medical Branch, Galveston, TX 77555, USA; 2 Department of Neuroscience and Cell Biology, University of Texas Medical Branch, Galveston, TX 77555, USA; 3 Sealy Center for Vaccine Development, University of Texas Medical Branch, Galveston, TX 77555, USA; 4 George and Cynthia Woods Mitchell Center for Neurodegenerative Diseases, University of Texas Medical Branch, Galveston, TX 77555, USA; 5 Rosensteil Basic Medical Sciences Research Center, Brandeis University, Waltham, Massachusetts, 02454, USA; 6 Barshop Institute for Longevity and Aging Studies, University of Texas Health Science Center, San Antonio, Texas 78229, USA

**Keywords:** Aggregation, Amyotrophic lateral sclerosis, Protein surface hydrophobicity, Superoxide dismutase, Heat shock factor 1

## Abstract

**Background:**

Mutations in the Cu/Zn superoxide dismutase gene (SOD1) are responsible for 20% of familial forms of amyotrophic lateral sclerosis (ALS), and mutant SOD1 has been shown to have increased surface hydrophobicity *in vitro*. Mutant SOD1 may adopt a complex array of conformations with varying toxicity *in vivo*. We have used a novel florescence-based proteomic assay using 4,4’-bis-1-anilinonaphthalene-8-sulfonate (bisANS) to assess the surface hydrophobicity, and thereby distinguish between different conformations, of SOD1and other proteins *in situ*.

**Results:**

Covalent bisANS labeling of spinal cord extracts revealed that alterations in surface hydrophobicity of H46R/H48Q mutations in SOD1 provoke formation of high molecular weight SOD1 species with lowered solubility, likely due to increased exposure of hydrophobic surfaces. BisANS was docked on the H46R/H48Q SOD1 structure at the disordered copper binding and electrostatic loops of mutant SOD1, but not non-mutant WT SOD1. 16 non-SOD1 proteins were also identified that exhibited altered surface hydrophobicity in the H46R/H48Q mutant mouse model of ALS, including proteins involved in energy metabolism, cytoskeleton, signaling, and protein quality control. Heat shock proteins (HSPs) were also enriched in the detergent-insoluble fractions with SOD1. Given that chaperones recognize proteins with exposed hydrophobic surfaces as substrates and the importance of protein homeostasis in ALS, we crossed SOD1 H46R/H48Q mutant mice with mice over-expressing the heat shock factor 1 (HSF1) transcription factor. Here we showed that HSF1 over-expression in H46R/H48Q ALS mice enhanced proteostasis as evidenced by increased expression of HSPs in motor neurons and astrocytes and increased solubility of mutant SOD1. HSF1 over-expression significantly reduced body weight loss, delayed ALS disease onset, decreases cases of early disease, and increased survival for the 25^th^ percentile in an H46R/H48Q SOD1 background. HSF1 overexpression did not affect macroautophagy in the ALS background, but was associated with maintenance of carboxyl terminus of Hsp70 interacting protein (CHIP) expression which declined in H46R/H48Q mice.

**Conclusion:**

Our results uncover the potential importance of changes in protein surface hydrophobicity of SOD1 and other non-SOD1 proteins in ALS, and how strategies that activate HSF1 are valid therapies for ALS and other age-associated proteinopathies.

## Background

It has been proposed that exposure of hydrophobic surfaces increases the propensity of non-native proteins to oligomerize and form aggregates in a wide range of age-associated neurodegenerative diseases including amyotrophic lateral sclerosis (ALS), Alzheimer’s (AD), Parkinson’s (PD), and Huntington’s diseases. ALS is the most common adult motor neuron disease characterized by progressive degeneration of motor neurons, which results in muscle atrophy and weakness, followed by paralysis and death. A number of different mutations in genes encoding Cu/Zn superoxide dismutase (SOD1) [1], TAR DNA binding protein 43 (TDP-43) [2], and 17 others are associated with familial forms of ALS (fALS), which make up 10% of total ALS cases. The first known genetic link to fALS was SOD1, which is responsible for 20% of familial cases, and over 150 mutations in SOD1 have been described [3]. SOD1 mutations can be grouped into two families based on their biophysical effects to the protein: pseudo-wild-type mutants which retain metal binding and enzyme activity, and metal binding region mutants such as H46R/H48Q, which have only partial to no metal binding ability and reduced or no enzyme activity [4,5]. Despite being responsible for the disproportionation of superoxide radicals to oxygen and hydrogen peroxide, mutations in SOD1 are known to be toxic due to a gain of function rather than a loss of function [6]. Increased surface hydrophobicity of SOD1 mutants is a common feature [7,8] and may be important in gain of functionality through a structure-function relationship, however the degree to which soluble mutant SOD1 is misfolded *in situ* is unknown and difficult to measure using conventional assays. Changes in protein surface hydrophobicity are important because exposure of hydrophobic domains may facilitate the formation of new protein-protein interactions and aggregation of proteins which are observed in all cases of ALS. If allowed to persist *in vivo*, surface exposed hydrophobic domains could lead to formation of oligomers or seeding of amorphous or fibrillar protein aggregates that correlate with cellular toxicity [9,10]. Importantly, substrate specificity of major heat shock proteins (HSPs) is dictated by sequence-specific hydrophobic amino acids that frequently occur in proteins but are usually buried and not surface-exposed [11-14]. Thus, exposed surface hydrophobicity is a significant recognition signal for HSP binding and subsequent re-folding or degradation by chaperones and co-chaperones via the ubiquitin proteasome system (UPS) [15] or autophagy [16].

Soluble, conformationally altered proteins and those with increased surface hydrophobicity are difficult to measure and screen for in biological samples without prior fractionation and purification, and available methods are not amenable to locating the specific domains where unfolding occurs. In addition, difficulties in replicating the off-pathway folding events that occur *in vivo*[17] make measuring conformationally altered species *in situ* advantageous. To address these difficulties, we have previously developed a novel fluorescence-based proteomic assay using 4,4’-bis-1-anilinonaphthalene-8-sulfonate (bisANS) that can detect changes in protein conformation on the basis of changes in protein surface hydrophobicity from soluble *in situ* tissue proteomes [18,19]. Using this assay we have found that changes in protein conformation do occur in skeletal muscle during ALS progression, experimental denervation, and muscle injury [18,20,21], and that the bisANS incorporation sites can be mapped onto proteins [21] for further targeting studies with conformation-specific antibodies [22], or other methods. In this study, we measure changes in surface hydrophobicity of proteins from the spinal cords of H46R/H48Q mice in order to examine the *in situ* surface hydrophobicity of soluble mutant SOD1 and non-SOD1 proteins from this model. By covalently labeling proteins with the conformation-sensitive dye bisANS, which fluoresces when it binds to apolar surfaces, we have found that the H46R/H48Q mutation in SOD1 provokes formation of high molecular weight SOD1 species with a lower solubility due to increased exposure of hydrophobic surfaces. Furthermore, we have uncovered changes in the surface hydrophobicity profile of 16 non-SOD1 proteins that are involved in energy metabolism pathways, cytoskeletal framework/cell mobility, signaling, and protein quality control systems.

Heat shock factor 1 (HSF1) is a 57 kDa member of the HSF family, and is the major regulator of HSP expression [23]. Given that HSPs are cytoprotective and recognize exposed surface hydrophobicity in their selection of substrates, HSF1 is an attractive pharmacological target. Several pharmacological activators of HSF1 are known, and function through inhibition of the proteasome or negative regulators of HSF1, such as HSP90. The hydroxylamine compounds bimoclomol and arimoclomol prolong the activation of HSF1. Arimoclomol was tested on the G93A mouse model of ALS and it was found to increase lifespan by 22% [24] and is currently in phase 2/3 clinical trials for ALS [25]. The arimoclomol treated mice had elevated levels of HSP70 and 90 compared to untreated G93A mice, suggesting that HSP expression through the HSF1 system was protective in ALS, however it is unknown whether metal binding region mutants will be protected by enhancing protein homeostasis. Riluzole, an FDA approved drug to treat ALS has been shown to increase latent HSF1 levels and enhance the heat shock response (HSR) [26,27]. Importantly, increasing levels of HSF1 by the use of transgenes [28] or through glutamine and the CAAT enhancer-binding protein-β (C/EBP-β) [29], are alternate ways to upregulate HSF1 and enhance the HSR due to titration of the HSF1 inhibitor HSP90. This is especially important for motor neurons, which are reported to have a high threshold for activation of HSF1[30]. Several studies have shown that the over-expression of HSF1 extends lifespan and protects against various types of pathologies. For example, in *C. elegans* over-expression of HSF1 extends lifespan [31], while its inhibition shortens lifespan. Activation of HSF1 using the HSP90 inhibitor 17-N-allylamino-17-demethoxygeldanamycin (17-AAG) led to an extension in lifespan of a drosophila model of ALS, owing to the upregulation of the drosophila ortholog of αB-crystallin [32]. We and others have demonstrated a protective role of HSF1 against protein misfolding and aggregation in other neurodegenerative diseases, including AD [33], Huntington’s disease [34], and prion diseases [35]. Taken together, these studies confer the beneficial effects of an HSF1-based ALS therapy and an important role of the HSF1-mediated HSR in protecting against ALS.

We have created a transgenic mouse that over-expresses human HSF1 (HSF1^+/0^) 2-4 fold in all tissues especially the CNS [28]. We have shown that HSF1^+/0^ mice have an enhanced HSR [28], and are protected from AD-like deficits in memory [36]. In the current study, the effect of HSF1 over-expression in a mouse model of ALS was examined and found to significantly delay loss of bodyweight, disease onset, early disease, and survival in the 25^th^ percentile suggesting that enhanced control of protein surface hydrophobicity by upregulating HSF1 is a potential target for the treatment of ALS and other proteinopathies.

## Results

### Soluble Mutant SOD1 in spinal cord extracts has increased surface hydrophobicity

**Figure 1:**
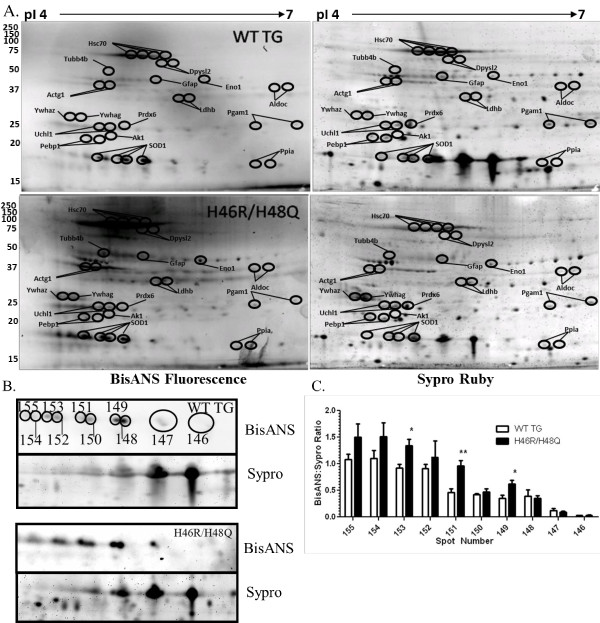
** Altered surface hydrophobicity of mutant SOD1 and non-SOD1 proteins in the spinal cords of symptomatic ALS mice. A)** Representative 2D gels of wild type transgenic human SOD1 (WT TG, n = 8) and H46R/H48Q (n = 12) with molecular weights (left axis) and isoelectric points (pI, upper axis). Spots that significantly differed from WT TG in hydrophobic ratio are circled and annotated based on the gene names of their accession numbers identified by MALDI-TOF mass spectrometry. **B)** Enhanced region of 2D gels containing WT SOD1 and H46R/H48Q mutant SOD1 proteins. BisANS fluorescence and corresponding total protein stained with Sypro Ruby are shown. Quantitated SOD1 spots are shown with numbered ellipses and correspond to the quantitated hydrophobic ratio shown in **C)**. Bars represent the mean hydrophobic ratio +/- standard deviation of 10- 8 mice per group. *p<0.05, **p<0.01 by one-way ANOVA.

In order to assess the global distribution of proteins with altered exposure of surface hydrophobicity in the spinal cords of symptomatic ALS mice, the soluble S1 fraction was labeled with bisANS and separated by 2D gel electrophoresis (Figure 1A). As shown in Figure 1B spots corresponding to human SOD1 were identified by MALDI-TOF mass spectrometry (Table 1) and further confirmed by Western blot using unlabeled spinal cord extracts as specific for SOD1 (Additional file 1: Figure S1). These spots specific for SOD1 were then quantitated for their bisANS fluorescence and normalized for protein by Sypro Ruby in order to determine their hydrophobicity ratio (Figure 1B and C). We observed that SOD1 separated into multiple spots with different isoelectric points, as previously shown by others in unlabeled extracts [37]. SOD1 spot numbers 153, 151, and 149 (Figure 1B&C) showed significant increases in the hydrophobic ratio compared to WT SOD1. Increased surface hydrophobicity of mutant SOD1 suggests that it may have increased propensities for aggregation and/or toxicity [38].

**Table 1:** Protein identification table and hydrophobic ratios

**Protein Name**	**Accession #**	**Mass Values Searched**	**Mass Values Matched**	**Mowse Score**	**Probability of false hit**	**Percent Sequence Coverage**	**Fold H46R/H48Q /WT TG Sypro**	**Fold H46R/H48Q /WT TG BisANS:Sypro**	**P value**
**Heat shock cognate 71 kDa protein (Hsc70)**	P63017	**44**	**15**	**108**	**2.3e-06**	**28**	**1.43 (0.06)**	1.37*	**0.0001**
		**36**	**13**	**86**	**4.1e-04**	**25**			
		**85**	**19**	**96**	**3.4e-05**	**33**			
		**102**	**25**	**110**	**1.4e-06**	**37**			
**Dihydropyrimidinase-related protein 2 (Dpysl2)**	O08553	**27**	**9**	**70**	**1.5e-02**	**19**	**1.33 (0.16)**	2.06*	**0.0080**
		**52**	**12**	**71**	**1.2e-02**	**24**	**1.24 (0.31)**	2.19*	**0.0256**
**Glial fibrillary acidic protein isoform 1 (Gfap)**	P03995	**87**	**24**	**148**	**2.3e-10**	**46**	**0.95 (0.32)**	1.91*	**0.0119**
**Tubulin beta-4B chain (Tubb4b)**	P68372	**45**	**13**	**97**	**2.6e-05**	**27**	**1.25 (0.33)**	2.08*	**0.0062**
**Alpha-enolase (Eno1)**	P17182	**37**	**18**	**183**	**7.2e-14**	**42**	**0.99 (0.98)**	3.08*	**0.0118**
**Actin, cytoplasmic 2 (Actg1)**	P63260	**76**	**16**	**101**	**1.1e-05**	**45**	**1.62 (0.01)**	**0.84**	**0.0467**
		**29**	**9**	**80**	**1.5e-03**	**30**	**1.63 (<.01)**	0.72*	**0.0020**
**Fructose-bisphosphate aldolase C (Aldoc)**	P05063	**33**	**15**	**138**	**2.3e-09**	**45**	**1.13 (0.65)**	**1.13**	**0.0128**
		**65**	**16**	**116**	**3.6e-07**	**46**	**0.96 (0.92)**	2.98*	**0.0189**
**L-lactate dehydrogenase B chain (Ldhb)**	P16125	**35**	**9**	**76**	**3.5e-03**	**20**	**1.00 (0.99)**	1.67*	**0.0284**
		**46**	**10**	**77**	**2.6e-03**	**28**	**1.11 (0.64)**	**1.21**	**0.7792**
**Phosphoglycerate mutase 1 (Pgam1)**	Q9DBJ1	**37**	**9**	**85**	**4.5e-04**	**46**	**1.64 (0.20)**	2.07*	**0.0136**
		**29**	**11**	**125**	**4.6e-08**	**52**	**1.42 (0.16)**	**1.14**	**0.6404**
**14-3-3 zeta (Ywhaz)**	P63101	**67**	**13**	**80**	**1.6e-03**	**35**	**3.25(<.01)**	0.35***	**0.0006**
**14-3-3 protein gamma (Ywhag)**	P61982	**55**	**12**	**78**	**2.3e-03**	**31**	**2.26 (<.01)**	0.47***	**0.0001**
**Peroxiredoxin-6 (Prdx6)**	O08709	**39**	**15**	**135**	**4.6e-09**	**53**	**1.48 (0.02)**	0.75*	**0.0442**
**Ubiquitin carboxy-terminal hydrolase L1 (Uchl1)**	Q9R0P9	**40**	**10**	**75**	**4.2e-03**	**42**	**2.28 (0.02)**	0.55*	**0.0141**
		**28**	**10**	**90**	**1.6e-04**	**42**	**2.11 (0.04)**	0.57**	**0.0006**
**Adenylate kinase isoenzyme 1 (Ak1)**	Q9R0Y5	**15**	**6**	**73**	**7.6e-03**	**37**	**1.33 (0.48)**	0.59*	**0.0113**
**Phosphatidylethanolamine-binding protein 1 (Pebp1)**	P70296	**15**	**6**	**82**	**8.1e-04**	**48**	**1.82 (0.04)**	0.66*	**0.0179**
		**12**	**5**	**70**	**1.5e-02**	**36**	**1.36 (0.20)**	**0.85**	**0.6637**
**Peptidyl-prolyl cis-trans isomerase A (Ppia)**	P17742	**56**	**18**	**113**	**7.2e-07**	**57**	**0.76 (0.37)**	**1.62**	**0.0356**
		**48**	**14**	**91**	**1.1e-04**	**40**	**1.11 (0.76)**	4.44**	**0.0106**
**Human Cu, Zn Superoxide Dismutase (SOD1)**	P00441	**40**	**8**	**76**	**7.0e-03**	**47**	**0.42 (<.01)**	1.75*	**0.0241**
		**14**	**6**	**80**	**2.2e-03**	**28**	**0.52 (0.04)**	**1.13**	**0.0335**
		**36**	**8**	**67**	**4.6e-02**	**34**	**0.45 (0.01)**	2.08**	**0.0051**
		**28**	**7**	**74**	**9.2e-03**	**38**	**0.63 (0.13)**	1.46*	**0.0046**

Protein IDs and gene names (in parentheses) are shown as well as fold-changes in their respective hydrophobic ratios and abundance with respect to matched WT TG spots. Statistical significance is indicated by asterisks *p = 0.05, **p = 0.01, ***p < 0.01 for ANOVA tests and p values of student’s t test are reported in parentheses.

### Non-SOD1 proteins with altered surface hydrophobicity in soluble fractions of spinal cord from H46R/H48Q mice

Since the toxic gain of function acquired by mutations in SOD1 may also alter non-SOD1 proteins in the spinal cord, we quantitated the non-SOD1 spots in the bisANS labeled extracts separated by 2D gels shown in Figure 1. We observed conformational alteration in a number of non-SOD1 proteins, and their fold changes in protein level and hydrophobicity ratio with respect to non-mutant WT mice were examined (Table 1 and Figure 1).

We examined one of the non-SOD1 proteins ubiquitin carboxyl hydrolase L1 (UCHL1) in more detail, due to its role in maintaining mono-ubiquitin pools and abundance in spinal cord. Changes in UCHL1 surface hydrophobicity were found to correlate with a dimeric state in recombinant human UCHL1 separated by size exclusion chromatography into monomeric and dimeric states (Additional files 2: Figures S2 and Additional files 3: Figure S3).

### BisANS docked to multiple sites around the metal binding region of H46R/H48Q SOD1

**Figure 2:**
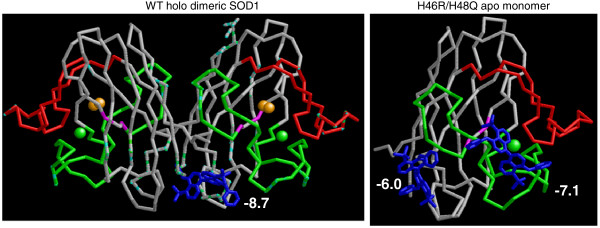
** BisANS docking with SOD1.** BisANS (blue) docked with WT human SOD1 [2C9V] and H46R/H48Q [3GQP]. The zinc loop (aa49-84) is colored green, and electrostatic loop (aa121-144) is colored red. Amino acids 46 and 48 are colored magenta, copper ions (orange), and zinc ions (green). Binding energies are given for bisANS binding sites in kcal/mol. H46R/H48Q possess a binding site for bisANS in the metal binding region, whereas holo WT dimeric SOD1 does not.

In order to investigate possible binding sites of bisANS in WT or mutant SOD1, crystal structures of dimeric WT holo human SOD1 [2C9V] and monomeric H46R/H48Q apo SOD1 [3GQF] were docked with bisANS (Figure 2), as WT holo SOD1 is known to exist as a dimer, while H46R/H48Q has been reported to be poorly metallated and can exist as a monomer [39,40]. As shown in Figure 2, bisANS (colored blue) had one energetically and geometrically favorable binding site between WT SOD1 monomers, but H46R/H48Q had multiple possible binding sites around the copper binding (colored green) and electrostatic loops (colored red) of SOD1, which have been reported to be disordered in H46R/H48Q SOD1[39]. These data were consistent with *in situ* data (Figure 1) showing that H46R/H48Q had a greater degree of exposed surface hydrophobicity than wild-type, likely due to instability and greater exposure of the metal binding region in SOD1.

### SOD1 and chaperones enriched in detergent insoluble fractions

**Figure 3:**
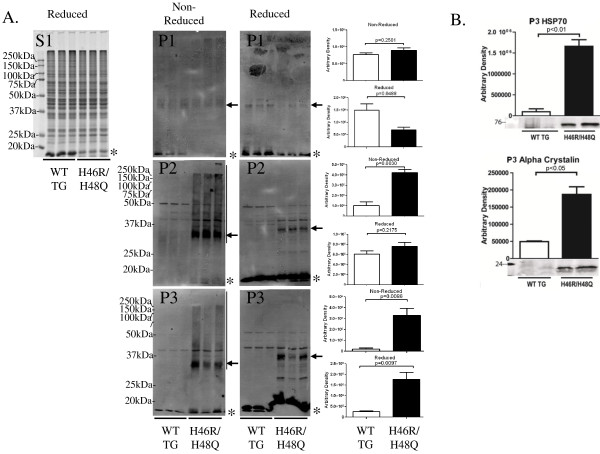
** Mutant SOD1 and chaperones co-fractionate in the detergent insoluble fractions.** Spinal cord extracts were subjected to differential detergent extraction and **A)** the soluble fraction was electrophoresed under reducing and denaturing conditions and stained with Coomassie (S1) and P1-P3 fractions were immunoblotted for SOD1 under non-reducing or reducing conditions (P1-P3). Asterisks indicate the position of the monomeric SOD1. High molecular weight reactivity to the SOD1 antibody was detected in the H46R/H48Q extracts but not WT TG (indicated by vertical bar). High molecular weight reactivity to SOD1 diminished with addition of a reducing agent, however a band corresponding to dimeric SOD1 persisted and is indicated by an arrow. **B)** Inducible heat shock protein 70 and αB-crystallin co-fractionated with mutant SOD1 in the detergent insoluble fractions of spinal cord. *p = 0.05, **p = 0.01, Bars represent the mean of 6 mice +/- SD.

In order to determine whether the solubility of mutant SOD1 correlated with the observed differences in hydrophobicity, we examined spinal cord extracts for the presence of SOD1 by differential detergent extraction (Figure 3A). In the Coomassie stained soluble S1 fraction, SOD1 migrates as a monomer under reducing and denaturing SDS page at 17 kDa, and differences in levels of SOD1 between WT TG and H46R/H48Q are affected by both transgene copy number and solubility. Mutant SOD1 formed high molecular weight bands in the P2 and P3 fraction (Figure 3A, arrow), and showed high molecular weight smearing corresponding to SOD1 (Figure 3A, vertical bar and asterisk), which was partially resolved by reducing the sample with dTT. In pellet 3, we also observed a dramatic increase in the level of chaperones such as HSP70 and αB-crystallin, suggesting that these chaperones were bound to mutant SOD1 as has been shown with other SOD1 mutants[41] (Figure 3B).

### Effects of over-expression of HSF1 in H46R/H48QxHSF1 mice

**Figure 4:**
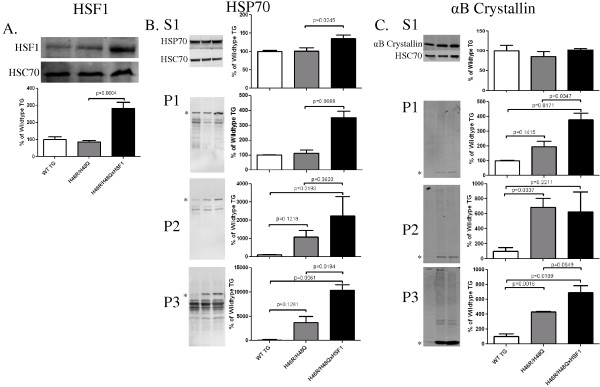
** Expression of HSF1 and distribution of HSPs in spinal cord. A)** Western blot of HSF1 in spinal cord homogenates from normal and symptomatic control mice normalized with HSC70, demonstrating the over-expression of HSF1 in H46R/H48QxHSF1 mice compared to H46R/H48Q littermates. **B)** Spinal cords were extracted with detergents of increasing ionic strength as described in the methods. S1 and P1-3 extracts were immunoblotted for inducible HSP70 **(B)** or αB-crystallin **(C)**. Asterisks are given at the position of the expected monomeric protein. p is indicated by brackets, bars represent the mean of 6 mice +/- SD.

**Figure 5:**
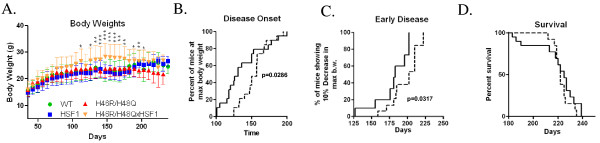
** Effect of HSF1 over-expression on body weight loss and healthspan of H46R/H48Q mice.** H46R/H48Q mice (Solid line) n = 20 and H46R/H48QxHSF1 (dashed line) n = 19. **A)** Beginning at 3 weeks of age, weekly averages of body weight are plotted for the survival cohort for WT (green circles), HSF1 (blue squares), H46R/H48Q (red triangles), and H46R/H48QxHSF1 (gold triangles). Asterisks indicate significant differences in H46R/H48QxHSF1 vs. H46R/H48Q at the *p<0.05, **p<0.01, and ***p<0.001 level. **B)** Disease onset was determined as the time animals reached their maximum bodyweight. **C)** Early disease was defined as a drop of 10% of the mouse maximal weight. **D)** Survival was defined as the point at which animals could not right themselves within 30s after being placed on their side.

**Figure 6:**
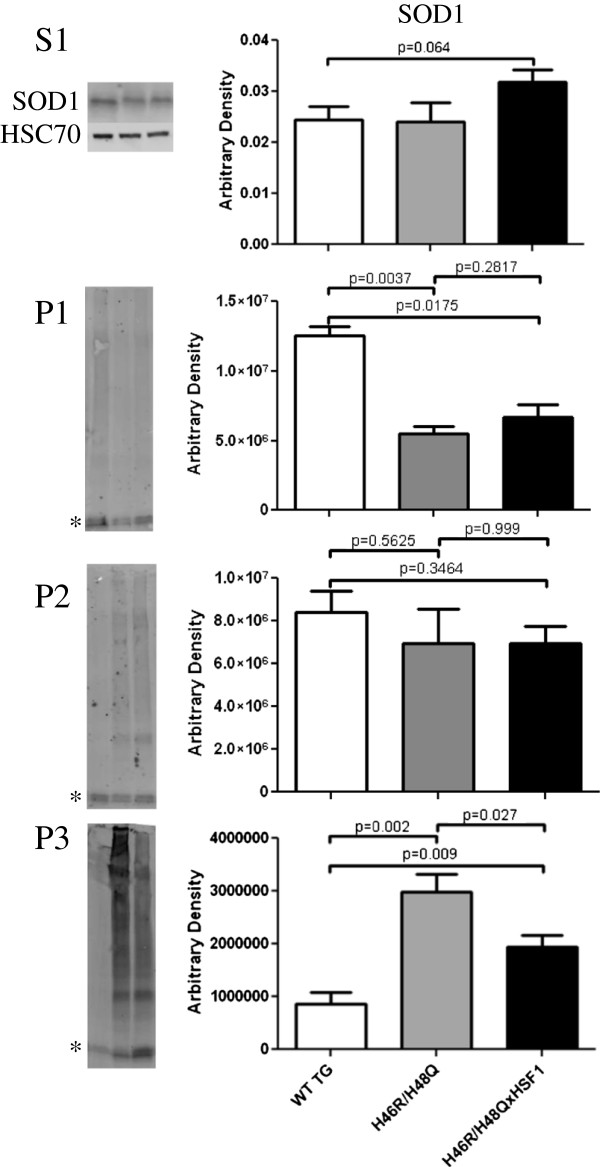
**Effect of HSF1 over-expression on mutant SOD1 solubility.** SOD1 in the soluble S1 and detergent soluble fractions P1-P3. Asterisks are given at the position of the expected monomeric SOD1. Bars represent an n = 6 +/- SD.

**Figure 7:**
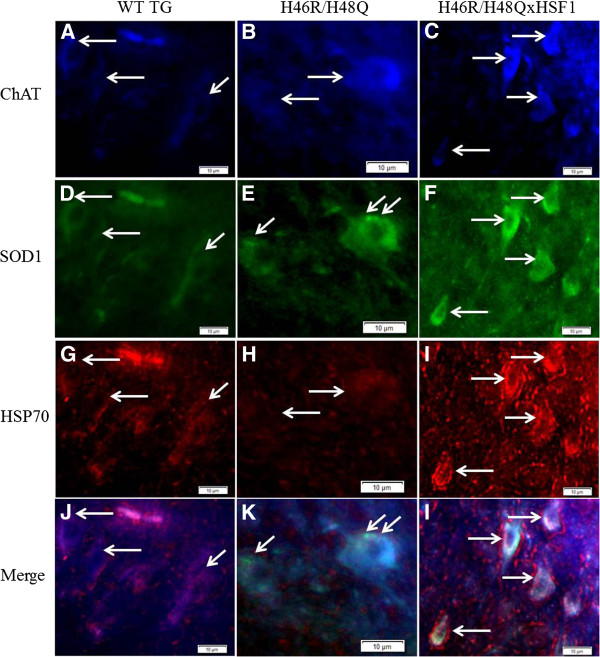
** Anterior horn region of lumbar spinal cord sections from mice at 220 days.** Motor neuron choline acetyltransferase (ChAT) positive cells **(A-C, arrows)** co-localized with SOD1 positive staining **(D-F, arrows)**. H46R/H48Q mice exhibited small intracellular peri-nuclear SOD1 reactive punctae **(E, arrows)**, while H46R/H48QxHSF1 tissues had a more even intracellular distribution of SOD1 staining **(F)**. This corresponded with differences in HSP70 distribution to SOD1 reactive punctae in H46R/H48Q mice **(H)** while in contrast, co-localization with ChAT reactive cells in H46R/H48QxHSF1 mice was stronger **(I)**. Scale bar represents 10 μm.

**Figure 8:**
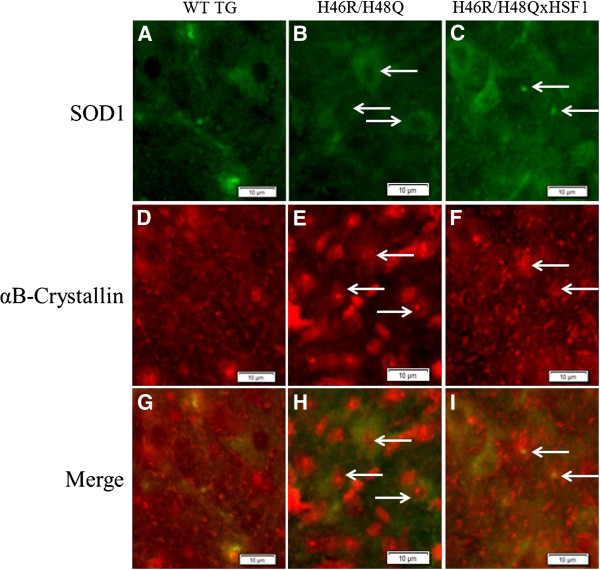
** Anterior horn region of lumbar spinal cord sections from mice at 220 days.** SOD1 staining **(A-C)** and small SOD1 positive punctae in H46R/H48Q vs. H46R/H48QxHSF1 tissues **(C, arrows)** co-localized diffusely with αB-crystallin throughout WT TG and H46R/H48QxHSF1 tissues **(D,F)**. Strikingly, αB-crystallin staining appeared more punctate and localized to cell nuclei of SOD1 expressing cells **(E,H, arrows and G-I)** in H46R/H48Q tissues. Scale bar represents 10μm.

Because chaperones and HSPs bind to exposed surface hydrophobic domains to assist protein re-folding or degradation, mutant H46R/H48Q SOD1 mice were crossed with the HSF1^+/0^ transgenic mice (H46R/H48QxHSF1) in order to determine the effects of HSF1 upregulation on disease progression of ALS. H46R/H48QxHSF1 were identified by PCR, and HSF1 over-expression was verified by Western blot of spinal cord extracts (Figure 4A) in mice at 222 days. HSF1 expression in the H46R/H48QxHSF1 symptomatic mice was three-fold higher (p = 0.0004) in the spinal cord compared to H46R/H48Q and WT TG littermates. Soluble levels of chaperones HSP70 (Figure 4B) but not αB-crystallin (Figure 4C) were elevated in the spinal cords of H46R/H48QxHSF1 mice, while levels of HSP70 and αB-crystallin were elevated in the P1 and P3 fractions of the spinal cord, indicating a more robust HSR in H46R/H48QxHSF1 mice. Differences in HSP70 and αB-crystallin could be detected in total homogenates as early as 197d in H46R/H48QxHSF1 compared to WT TG controls (Additional file 4: Figure S4). Disease progression can be followed by weight loss, and has been reported as a reliable marker of healthspan, as well as symptom and disease onsets in ALS mouse models. Hence, the body weights for H46R/H48Q and H46R/H48QxHSF1 cohorts were followed. H46R/H48QxHSF1mice (n=19) maintained body weights significantly better overall when compared to H46R/H48Q mouse littermates (n=20) (Figure 5A). Disease onset, the period of time when mice reached their maximum body weight, was also significantly delayed by over-expression of HSF1 (p=0.0286, Figure 5B). Initiation of early disease was calculated for H46R/H48QxHSF1 and H46R/H48Q littermates using body weights as described in the Methods. Compared to the H46R/H48Q littermates, the percentage of H46R/H48QxHSF1 mice that underwent early symptoms of the disease was significantly delayed (p=0.0317) compared to H46R/H48Q (Figure 5C). While overall survival was unaffected (Figure 5D), survival of the 25^th^ percentile was significantly different (p=0.017). Ubiquitous over-expression of HSF1 protected H46R/H48Q against ALS as evidenced by their improved body weight retention and delayed disease onset, symptom onset, and early survival. Also, over-expression of HSF1 led to a non-significant increase (p=0.064) in soluble mutant SOD1 and significantly reduced its levels in detergent insoluble fractions by 34% (p=0.027) (Figure 6). These data suggest that overexpression of HSF1 may have altered the solubility of SOD1 and improved protein homeostasis in motor neurons. To examine this, spinal cords were sectioned and the lumbar region was examined (Figure 7). As shown, the distribution of SOD1 in motor neurons was altered by overexpression of HSF1, as choline acetyltransferase (ChAT) positive motor neurons contained fewer SOD1 puncta and exhibited a more uniform staining for SOD1 in cell bodies compared to H46R/H48Q. This corresponded to a more intense staining for HSP70 in ChAT positive motor neurons compared to H46R/H48Q tissues. Likewise, αB-crystallin staining showed a striking change in its distribution in the H46R/H48Q tissues going from a diffuse pattern as seen in WT TG tissues to a more punctate nuclear pattern as seen in the large SOD1 positive cell bodies in the H46R/H48Q spinal cord (Figure 8). Overexpression of HSF1 appeared to restore this shift to resemble the appearance of WT TG. In addition to motor neurons, GFAP positive astrocytes also contributed a major portion of the HSP70 and αB-crystallin staining (Additional file 5: Figure S5).

**Figure 9:**
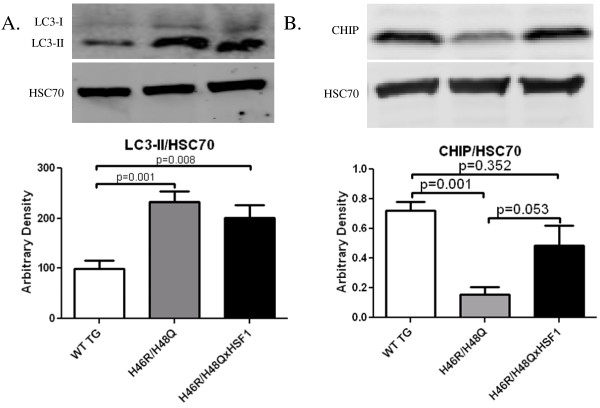
** Effects of HSF1 over-expression on measures of protein quality control in ALS**. Whole spinal cords were homogenized in 2%SDS and immunoblotted for **A)** LC3-II or **B)** CHIP and normalized with Hsc70. Bars represent an n = 6 +/- SD.

One possible explanation for the restoration of SOD1 solubility in tissues of H46R/H48QxHSF1 mice could be explained by enhanced chaperone-mediated turnover of mutant SOD1. Mutant SOD1 has been shown to be degraded by both the proteasome and macroautophagy [42]. Since HSF1 could affect induction of macroautophagy, we next examined levels of membrane-bound microtubule-associated proteins 1A/1B light chain 3A (LC3-II). Levels of LC3-II protein remained elevated in H46R/H48QxHSF1 mice as observed in H46R/H48Q (Figure 9A), while normalized levels of p62 were also unchanged by HSF1 overexpression (Additional file 3: Figure S3B) indicating that rates of macroautophagy were not affected. The carboxyl terminus of Hsp70 interacting protein (CHIP) is an important co-chaperone that has been shown to play a role in the polyubiquitination and proteasomal degradation of mutant SOD1 when bound to Hsp/Hsc70 [43]. To determine whether HSF1 over-expression would enhance CHIP expression, the expression levels of CHIP in the spinal cords from H46R/H48QxHSF1 mice were examined by Western blot. Levels of CHIP were significantly decreased (p=0.001) following mutant SOD1 over-expression, likely due to a shift in protein turnover in ALS away from the UPS, which was prevented by over-expression of HSF1 (Figure 9B).

## Discussion

A change in protein surface hydrophobicity can be an important indicator to determine alterations in protein structure, and thus indicative of losses or toxic gain of protein function. Exposure of protein surface hydrophobicity and disordered regions in proteins have been reported to precede aggregation [38], and to be an important intrinsic quality of proteins that can determine whether they are aggregation prone or capable of forming amyloid using bioinformatic approaches [10]. In addition to intrinsic hydrophobicity, proteins expose hydrophobic residues on their surface through post-translational modifications, mutations, or oxidative damage, which may not be predicted with bioinformatic approaches alone. SOD1 contains a greek key β-barrel which can be exposed or unfold due to SOD1 mutations near the metal binding region, and conformation-specific antibodies targeting this site can detect SOD1 in neuronal tissues of human familial and sporadic ALS patients [44,45]. The bisANS related compound ANS has similar properties to bisANS and has been used to show that the point in time at which amyloidogenic proteins exhibit their greatest toxicity correlates with a surge in surface hydrophobicity [46]. Increased surface hydrophobicity of the SOD1 mutant studied here *in situ* and by others has been described using different *in vitro* techniques [7,8,17,47]. In this proteomic study, we have utilized a covalent photolabeling approach with the hydrophobic dye bisANS to monitor the surface hydrophobicity of mutant SOD1 and other non-SOD1 proteins in the spinal cord of H46R/H48Q mice.

Consistent with several studies looking at the hydrophobicity of G93A or single mutant H46R or H48Q SOD1 using similar hydrophobic dyes *in vitro*[38,46], we observed alterations and an overall increase in soluble SOD1 surface hydrophobicity of previously reported isoelectric species of SOD1 [37] in the H46R/H48Q double mutant of SOD1 *in situ*. Our *in situ* analysis of the conformationally altered proteome shows for the first time only a fraction of the total SOD1 soluble pool exhibits these properties, and that the degree of exposed surface hydrophobicity is varied among isoelectric species of SOD1. These differences in surface hydrophobicity are important, and could reflect different conformational states that each mutant undergoes *in vivo* during symptomatic stages of ALS, metallation status of the protein [5], or may identify species of SOD1 that are most toxic. We found that the metal binding region appeared to be the most plausible target of bisANS in mutant SOD1 (Figure 3), and this can be confirmed by identification of the specific bisANS incorporation site(s) using mass spectrometric methods in future studies.

The surface hydrophobicity detected by bisANS correlated with the insolubility of mutant SOD1 and the presence of high molecular weight species of SOD1 in detergent insoluble fractions, which was partially dependent upon disulfide bonds or mixed disulfides. While the species of mutant SOD1 detected here with bisANS were soluble species, the conformational alterations in SOD1 alluded to by changes in surface hydrophobicity may be indicative of toxic gain of function. Interestingly, increases in surface hydrophobicity of WT SOD1 as detected by ANS correlate with a gain in SOD1’s ability to bind, cleave DNA or RNA, and induce its aggregation [47,48]. Mutant SOD1 has also been shown to display increased binding to neurofilament (NFL) mRNA [49], and to alter RNA homeostasis by binding to Hu antigen R and T-cell internal antigen 1 (TIA1) related protein [50], two key players in modulating stress granule formation and mRNA stability along with TDP-43. However it is unknown whether the increased hydrophobicity associated with the SOD1 mutants studied here enhances these reported gains of function in SOD1. Further studies are required to elucidate the direct impact that increased protein surface hydrophobicity has on toxic gains of function in SOD1. Aggregates in spinal cords of ALS patients as well as in the mice used in this study have been reported to contain various chaperones such as HSP70 and αB-crystallin [41], intermediate filament proteins, and others [51], supporting the idea that interventions that enhance proteostasis might be beneficial in ALS.

Since SOD1 mutations and aggregation are only observed in some ALS cases, there is good reason to suspect that the toxic effects of mutant SOD1 may be indirect and involve non-SOD1 proteins. For this reason, we also examined the changes in surface hydrophobicity of soluble non-SOD1 proteins in the spinal cords from symptomatic ALS mice. We found sixteen non-SOD1 proteins with significantly altered surface hydrophobicity, and these could be separated into groups such as energy metabolism, cytoskeletal organization, protein quality control, and signaling based upon their reported functions.

Although too numerous to discuss all, a few of the proteins in Table 1 deserve some discussion. Several glycolytic proteins are “moonlighting proteins” and perform functions outside of glycolysis with implications for neurodegenerative disease [21,52]. Changes in surface hydrophobicity in glycolytic enzymes correlate well with clinical and experimental observations that defects in energy homeostasis are a common feature in subsets of ALS patients [21,53,54]. In our study, we detected alterations in surface hydrophobicity of several glycolytic enzymes and proteins important in energy homeostasis including alpha-enolase, fructose-bisphosphate aldolase C, L-lactate dehydrogenase B chain, phosphoglycerate mutase 1, and adenylate kinase 1.

Alexander disease is a neurodegenerative disease caused by mutations in the glial fibrillary acidic protein (GFAP), leading to aggregates of GFAP and chaperones called Rosenthal fibers [55]. We detected significant increases in the surface hydrophobicity of GFAP, but whether the changes in GFAP hydrophobicity observed in this study were indicative of aggregation in ALS is unknown.

Defects in protein homeostasis and quality control are hypothesized as important mechanisms in neurodegenerative diseases [56]. The constitutive chaperone Hsc70, which is involved in several aspects of protein homeostasis, has been shown to bind to mutant SOD1 via interaction with CHIP to promote mutant SOD1 degradation, and is found in spinal cord aggregates from both mice [41] and humans [51]. In this study, surface hydrophobicity of Hsc70 in H46R/H48Q was significantly increased compared to WT transgenic littermates. This is consistent with the finding that increases in ANS binding are associated with thermal denaturation of Hsc70 [57]. Another chaperone, peptidyl-prolyl cis trans isomerase A (PPIA) also known as cyclophilin A, is an immunophilin that binds cyclosporine A and catalyzes the conversion of peptidyl-prolyl bonds from cis to trans. Over-expression of cyclophilin A has been found to protect cells from mutant SOD1-induced cell death [58], and to aggregate with Hsc70 and 14-3-3 gamma in G93A mice [51]. It is plausible that the alterations we observed in surface hydrophobicity correlate with these activities of cyclophilin A, although it has not been directly tested.

UCHL1 is an abundant protein in motor neurons, and deletions in the 7^th^ and 8^th^ exons cause gracile axonal dystrophy in mice, characterized by motor/behavior abnormalities and shortened lifespan [59]. UCHL1 is a monoubiquitin binding protein with weak ubiquitin hydrolase activity that may function in maintaining monoubiquitin pools [60]. UCHL1 has been shown to form dimers that enhance a ubiquitin-ubiquitin ligase activity [61]. In the same study this activity was associated with increased aggregation of α-synuclein in a cell model system of PD. Thus, it would appear that dimerization of UCHL1 promotes a gain of function activity that is detrimental under certain circumstances. In our study, we observed a decrease in surface hydrophobicity of UCHL1 (Figure 1, Table 1) that correlated with a similar decrease in surface hydrophobicity of UCHL1 between monomeric and dimeric forms *in vitro* (Additional file 2: Figure S2A and B). These data suggest that in ALS dimerization of UCHL1 is favored. Whether decreased surface hydrophobicity of UCHL1 translates into increased ubiquitin-ubiquitin ligation, or local depletion of the monoubiquitin pool remains to be determined.

The surface hydrophobicity of the signaling proteins 14-3-3 gamma and zeta isoforms were found to be significantly decreased. Originally highlighted in the brain for their roles in activating tryptophan and tyrosine hydroxylases, 14-3-3 proteins were later proven to have diverse functions mostly as scaffolding proteins, and have been implicated in a variety of neurodegenerative diseases [62]. With regards to ALS, 14-3-3 proteins have been shown to modulate NFL mRNA stability [63] and assist in targeting misfolded proteins to aggresomes by linking Bcl-associated athanogene 3 (BAG3) to histone deacetylase 6 (HDAC6) [64,65]. A triple phospho-mimetic mutant S58E/S184E/T232E has been linked to decreased bisANS incorporation in purified human 14-3-3 zeta [66]. Phosphorylation of Serine 58 in 14-3-3 zeta has been characterized as a molecular switch for induction of apoptotic cell death [67]. Therefore, in addition to its solubility being affected in ALS mice [51], changes observed in surface hydrophobicity of 14-3-3 in this study may be indicative of its phosphorylation.

Misfolded proteins are associated with exposed surface hydrophobicity, and the proteins that we identified with altered hydrophobicity likely represent metastable proteins that are easily misfolded during proteotoxic stress. Strikingly, Xu et al recently examined proteins that became insoluble in the brains of mice expressing mutant forms of amyloid precursor protein[68]. Many of the same proteins that we found misfolded here in the spinal cord of ALS mice were also found to be insoluble in the brains of mice modeling AD, supporting a connection to the misfolding we have measured using bisANS and the downstream aggregation process that occurs during proteotoxic stress. Misfolded proteins and proteins with exposed hydrophobicity are recognized by chaperones on the basis of surface hydrophobicity. Since we observed increases in surface hydrophobicity of mutant SOD1 and 16 other non-SOD1 proteins in the spinal cord of H46R/H48Q mice, we set out to determine whether over-expression of HSF1 would be protective against SOD1 mutant-mediated ALS and alteration in proteome surface hydrophobicity. As expected, over-expression of HSF1 was detected in the spinal cord of H46R/H48QxHSF1 mice by Western blot, and led to an increase in the solubility of SOD1. HSF1 over-expression led to a robust induction of HSP70 and αB-crystallin as detected in some soluble and insoluble fractions which could be detected as early as 197 days. However, HSP induction was not able to completely reverse SOD1 aggregation. Co-localization of HSPs by immunofluorescence and increased co-fractionation of HSP70 and αB-crystallin with SOD1 in insoluble fractions measured here, and the observations that these HSPs bound to mutant SOD1 *in vivo* by others [41] suggests they were associated with aggregated SOD1. Immunofluorescence of H46R/H48Q mice also detected an increased number of cells with nuclear localized αB-crystallin staining and speckles that were absent in double transgenic mice (Figure 8E, H). Recently nuclear localization of αB-crystallin has been shown to be associated with stress and associates with the survival of motor neuron complex[69]. Association of αB-crystallin with alpha 7 subunits of the 20S proteasome suggest it could also be involved in facilitating turnover of bound aggregates [70]. This is supported by the observations that differences in protein homeostatic machinery were detected in the UPS associated co-chaperone CHIP, but not macroautophagy of H46R/H48QxHSF1 mice. ALS is a multifactorial disorder affecting multiple cell types and physiological phenotypes, and these are reflected in transgenic mouse models of ALS[71]. H46R/H48QxHSF1 maintained a 3-fold increase in HSF1 expression throughout the study, which led to retention in body weight and delay in disease onset, early disease, and survival of the 25^th^ percentile with exception of overall survival of the experimental cohorts (Figure 5). Motor neuron death is an early event in the disease process, and is responsible for the timing and early symptoms in ALS [72], while astrocytes[73] and microglia [74] are responsible for disease progression suggesting that HSF1 over-expression had a primarily beneficial effect on motor neurons, but not on astrocytes or microglia.

## Conclusions

We show that changes in surface hydrophobicity as detected by bisANS relate to important gains or losses of function in soluble SOD1 and the non-SOD1 proteins we identified. Importantly, this study demonstrates that soluble fractions of mutant SOD1 are indeed misfolded and exhibit increased surface hydrophobicity, which correlates with the insolubility of mutant SOD1 and may be indicative of toxic gain of function. In future studies, mapping the bisANS incorporation sites to the isoelectric species of mutant SOD1 and the other non-SOD1 proteins that were altered will enable these domains to be targeted in order to determine their possible roles in ALS. Furthermore, the bisANS fluorescent-based assay used here can be applied as part of a high-throughput screening approach to identify compounds/drugs that interfere with the exposure of hydrophobic surfaces of metastable proteins or prevent aggregation. Over-expression of HSF1 was associated with maintenance in bodyweight, delayed disease onset, progression, and early survivorship. These data indicate that there is a functional and survival benefit to over-expression of HSF1 and enhancing the heat shock response, and validates therapeutic strategies aimed at activating HSF1 for ALS models. This is the first study to demonstrate that over-expression of full-length HSF1 is beneficial in ALS, and further shows that increasing HSF1 protein may be an additional strategy to activate HSF1 rather than by inducing proteotoxic stress or inhibiting HSP90.

## Methods

### Mice

Mice were housed in a temperature and humidity-controlled vivarium at the Audie L. Murphy VA Hospital. Wild type transgenic human SOD1 (WT TG) mice were backcrossed to C57B6/J mice 4 times. The SOD1 H46R/H48Q mutant mice (H46R/H48Q) were generated by David Borchelt (line 139) [75] on a C3HeJ B6 background and backcrossed over 10 generations to a C57BL/6J background in our facility. HSF1^+/0^ were derived and maintained on a C57BL/6 background, and the generation of these mice has been described previously [28]. Male H46R/H48Q breeders were mated with female HSF1^+/0^ mice to generate mice for the survival cohorts, and the lifespans of the male breeders were monitored to ensure they did not deviate from parental lines due to mutant SOD1 copy loss. Offspring were genotyped to obtain double transgenic mice that contained both mutant H46R/H48Q and the HSF1 transgene (H46R/H48QxHSF1). Genotyping was performed by PCR using primers specific for the human SOD1 gene [1] and as previously described for the human HSF1 transgene [28]. Body weight measurements began when mice reached the age of 120 days and recording was performed bi-weekly until death (n=19/genotype). Disease onset was calculated as the day a mouse reached its maximal bodyweight, while early disease is defined as the day at which a 10% decrease in its maximal bodyweight was observed, and survival is determined at the day at which a mouse is unable to right itself when place on its side for 30s [76,77]. All procedures for handling animals in this study were reviewed and approved by the Institutional Animal Care and Use Committee (IACUC) of the Audie L. Murphy Memorial Veterans Hospital.

### BisANS assay

Covalent UV photolabeling of bisANS to proteins was performed essentially as described [21]. Briefly, soluble fractions (S1) of spinal cord tissue were isolated on ice by homogenization of tissue with 5 volumes of bisANS labeling buffer (50 mM Tris 10 mM MgSO_4_ pH 7.4 plus protease inhibitors) followed by centrifugation at 100,000*g* for 1hr at 4°C. It has been shown that incubation of proteins with high dye:protein ratios of 100:1 or more can induce conformational changes in some proteins [78]. Therefore in all comparisons, we labeled 1 mg/ml solutions of cytosolic protein with 200 μM bisANS with a handheld 365 nm UV lamp (UVP, Upland, CA) for 30 minutes on ice in a 96-well plate with non-mutant SOD1 over-expressing mice as controls. At this protein concentration it was estimated that the dye:protein ratio was approximately 8:1 based on abundance and molecular weights of Sypro Ruby spots in 2D gel profiles. BisANS labeled proteins were then precipitated 1:1 with 20% trichloroacetic acid. Precipitates were centrifuged at 4°C at 18,000*g* for 30 min, and pellets were washed twice by disrupting with ice cold 1:1 ethanol:ethyl acetate. Cleaned pellets were dried and then dissolved in 6M urea, 4% CHAPS, 0.4% ampholytes. For SDS PAGE gels, 15 μg of protein was diluted in 6X laemmli buffer and separated on 15% polyacrylamide gels. Following electrophoresis, gels were imaged for bisANS and stained with Sypro Ruby.

### 2D gel electrophoresis

Following determination of protein concentration by BCA assay, 4 μl of destreak reagent (GE Health) was added to 200 μg of bisANS labeled protein, which was focused on 4-7 immobilized pH gradient (IPG) strips for 12 hr. Focused IPG strips were then washed in equilibration buffer (50 mM Tris buffer pH 8.8, 6M urea, 30% glycerol, 2% SDS) containing 10 mg/ml dithiothreitol (dTT) for 15 min followed by 25 mg/ml iodoacetamide. Equilibrated strips were then separated on 15% polyacrylamide gels, placed in 10% methanol at 4°C, and visualized for bisANS fluorescence on a UV imager with a 365 nm illumination source for 30 seconds (AlphaImager). 2D gels were placed in fixing solution (10% methanol 7% acetic acid) for 1hr and then stained in Sypro Ruby overnight with gentle agitation. Following destaining with fixing solution, gels were placed in doubly deionized water for 1 hr and then imaged for Sypro Ruby fluorescence on a Typhoon 9410 imager (GE Health). BisANS and corresponding Sypro Ruby spots were matched and quantitated by densitometry using Imagequant (Molecular Dynamics). Hydrophobic ratios were determined by dividing the bisANS densitometric value by corresponding Sypro Ruby densitometric values. The mean hydrophobic ratios for each group were then compared using one-way ANOVA. Spots with significantly different hydrophobic ratios were picked using an ExQuest spot cutter (Biorad).

### Mass spectrometry

Picked spots were digested in gel with sequencing grade trypsin (promega) overnight at 37°C. Peptide digests were then spotted to a target with α-cyano-4-hydroxycinnamic acid, and identified by matrix-assisted laser desorption ionization time of flight (MALDI-TOF) mass spectrometry (Voyager STR, Applied Biosystems). Peak lists were generated by Data Explorer version 4.0.0.0 (Applied Biosystems) using advanced baseline correction peak width parameters of 32, flexibility of 0.5, and degree of 0.1; noise filter parameter of 0.7; gaussian smooth parameter of 5; noise reduction parameter of 2.00. Trypsin autocatalytic peaks were used to internally calibrate spectra, and were excluded from peak lists for Mascot database searches. Peptide mass fingerprints were then searched using Mascot (Matrix Science) and the NCBInr 10/01/2012 database allowing 1missed cleavage site. Variable modifications of carbamidomethylated cysteine and oxidation of methionine were included and a peptide mass tolerance of 100 ppm was used.

### Western blotting

Insoluble material remaining from homogenized spinal cords was sequentially extracted with detergents of increasing ionic strength as described previously [21]. In brief, after centrifugation of tissue homogenates at 100,000*g* the pellet was sonicated in bisANS labeling buffer to remove cytosolic proteins and re-pelleted at 100,000*g* for 30 min. Next, pellets were extracted in the same buffer containing 0.5% Nonidet P40 by sonication and re-pelleted at 100,000*g* for 30 min. This supernatant was termed P1. The remaining pellet was then sonicated in P1 buffer containing 0.25%SDS and 0.5% deoxycholate to isolate the P2 fraction, then P1 buffer containing 2% SDS and 0.5% deoxycholate to isolate the P3 fraction. Protein extracts were then boiled in non-reducing or reducing Laemmli buffer where indicated, and separated by 15% SDS PAGE followed by a 2hr wet transfer at 100V to polyvinylidine fluoride membranes (Biorad). Membranes were blocked in 5% milk Tris-buffered saline containing tween 20 (TBST) containing 20mM Tris, 137mM NaCl, and 0.4% tween 20 for 30 min at room temperature. Membranes were incubated with sheep anti-human SOD1 (1:1000, Calbiochem), mouse anti-UCHL1 (1:5,000 Abnova#H00007345-M01), mouse anti-Hsp70 (1:10,000, Stressgen; SPA-810), rabbit anti-HSF1 (1:10,000 dilution, Stressgen;SPA-901), heat shock cognate 70 (Hsc70) (1:20,000 dilution, Stressgen SPA-815), rabbit anti-LC3 (1:2,500 dilution, Cell Signaling#2775), rabbit anti-CHIP (1:1000, Cell Signaling#2080), rabbit anti-αB-crystallin (1:2000, Abcam #ab13497), overnight at 4°C with gentle agitation.

### BisANS docking

BisANS was docked to SOD1 (dimeric WT metallated: [2C9V] or monomeric apo H46R/H48Q:[3GQF]) using Autodock Vina [79]. Docked pdb files were generated and rendered by RasMol version 2.7.5.2.

### Immunofluorescence Microscopy

Mice were perfused transcardially with PBS and spinal cords were excised and fixed in PBS containing 4% paraformaldehyde at 4°C until processing. Lumbar regions of the spinal cord were imbedded in optimal cutting temperature compound and sectioned at 30 μm and affixed to poly-D-lysine coated slides. Sections were blocked in 4% goat serum in TBST and stained with choline acetyltransferase (1:250), glial fibrillary acidic protein (1:500), SOD1 (1:500) Heat shock protein 70 (1:500), or αB-crystallin (1:500) overnight at 4°C in 1% goat serum in TBST. Primary antibodies were visualized with secondaries conjugated with Alexa 350, 488, or 594 at 1:250 in 1% goat serum in TBST. Slides were imaged with an Olympus IX51 fluorescence microscope.

### Statistical analysis

2D gel data were analyzed for significance using one-way ANOVA. Post hoc tests were performed using Tukey’s test at a significance value of 0.05. For those spots with significantly different hydrophobic ratios compared to WT TG, the mean Sypro Ruby intensity and Western blot densitometric analyses were compared between WT TG and H46R/H48Q using a student’s t test. Disease onset, early disease, and survival curves were compared using the log-rank test (Wilcoxon).

## Abbreviations

SOD1: Cu/Zn superoxide dismutase 1; ALS: Amyotrophic lateral sclerosis; UCHL1: Ubiquitin carboxy-terminal hydrolase L1; bisANS: 4,4’-dianilino-1,1’-binaphthyl-5,5’-disulfonic acid; TDP-43: TAR DNA binding protein 43; FUS: Fused in sarcoma; fALS: familial ALS; HSPs: Heat shock proteins; HSF1: Heat shock factor 1; BCA: Bicinchoninic acid; ANOVA: Analysis of variance; TBST: Tris-buffered saline containing tween-20; dTT: dithiothreitol; HSP70: Inducible heat shock protein 70; Hsc70: Heat shock cognate 70; CHIP: Carboxyl terminus of Hsp70-interacting protein; actg1: cytoplasmic actin 2; aldoc: fructose bisphosphate aldolase C; ak1: adenylate kinase 1; pebp1: phosphatidylethanolamine-binding protein 1; ppia: peptidyl prolyl isomerase A; dpysl2: dihydropyrimidinase-related protein 2; gfap: glial fibrillary acidic protein isoform 1; tubb4b: tubulin beta-4B chain; eno1: alpha enolase; ldhb: lactate dehydrogenase B-chain; pgam1: phosphoglycerate mutase 1; Prdx6: Peroxiredoxin 6; Ywhaz: 14-3-3 protein zeta; Ywhag: 14-3-3 gamma; WT: Wild type; ChAT: Choline acetyltransferase; ANS: 1-Anilinonaphthalene-8-Sulfonic Acid; TIA1: T Cell intracellular antigen-1; BAG3: Bcl-associated athanogene 3; HDAC6: Histone deacetylase 6; MALDI-TOF: Matrix assisted laser desorption ionization time of flight; LC3-II: Membrane-bound microtubule-associated proteins 1A/1B light chain 3A.

## Competing interests

The authors declare that they have no competing interests.

## Authors’ contributions

SMS provided monomeric and dimeric UCHL1 protein. WKK performed UCHL1 experiments. OF performed bisANS docking, CU performed Western blotting. AP and PYL performed all other experiments, analyzed the data, and wrote the manuscript. All authors read and approved the final manuscript.

## Supplementary Material

Additional file 1: Figure S1 Western blot for SOD1 on 2D separated spinal cord. Spinal cords from WT TG mice were homogenized and unlabeled proteins were separated by 2D gel electrophoresis according to Methods. 2D gels were then equilibrated and blotted for human SOD1. SOD1 immunoreactive spots were matched with 2D gel spots for SOD1. Click here for file

Additional file 2: Figure S2 Surface hydrophobicity of monomeric and dimeric UCHL1. A) Recombinant monomeric (M) and dimeric (D) UCHL1 were fractionated by size exclusion chromatography and labeled with 5-molar excess bisANS and resolved by 12% SDS page, with unlabeled UCHL1 as a control. B) The resultant bisANS fluorescence in monomeric and dimeric preparations of UCHL1 were quantitated and normalized by Sypro Ruby staining. *p<0.05 by Student’s t test. Click here for file

Additional file 3: Figure S3 Recombinant UCHL1 UCHL1 protein was prepared essentially as described [80] with the following modifications: the induced cell pellet was resuspended in 4 mL PBS buffer/L culture prior to chromatography. Cells were disrupted by passage twice through a French pressure cell (Thermo Fisher) at 12,000 psi. Two peaks containing UCHL1 eluted from the final S200 chromatography step; the earlier peak was shown to correspond to the dimer by analytical gel filtration and native PAGE, whereas the latter peak was consistent with the monomer. Click here for file

Additional file 4: Figure S4 Effect of HSF1 overexpression on Chaperone and p62 levels in H46R/H48Q mice. Whole spinal cords were homogenized in 2%SDS and immunoblotted for the A) HSP70 and αB-crystallin or B) p62 and normalized with Hsc70. Bars represent an n=6 +/- SD. Click here for file

Additional file 5: Figure S5 Double immunofluorescence labeling of anterior horn lumbar spinal cord. H46R/H48QxHSF1 tissues were stained with HSP70 (red) and astrocyte marker Glial Fibrillary Acidic Protein (GFAP, blue). Some of the tissue staining for HSP70 and αB-crystallin can be accounted for by astrocytes as shown by colocalization with GFAP. Scale bar represents 10 μm. Click here for file
