# Supplementary figures and images for: Heat shock factor 1 over-expression protects against exposure of hydrophobic residues on mutant SOD1 and early mortality in a mouse model of amyotrophic lateral sclerosis
Source: Mol Neurodegener. 2013 Nov 21;8:43. doi: 10.1186/1750-1326-8-43 (PMC3907013; doi:10.1186/1750-1326-8-43)

Supplementary Figure 1

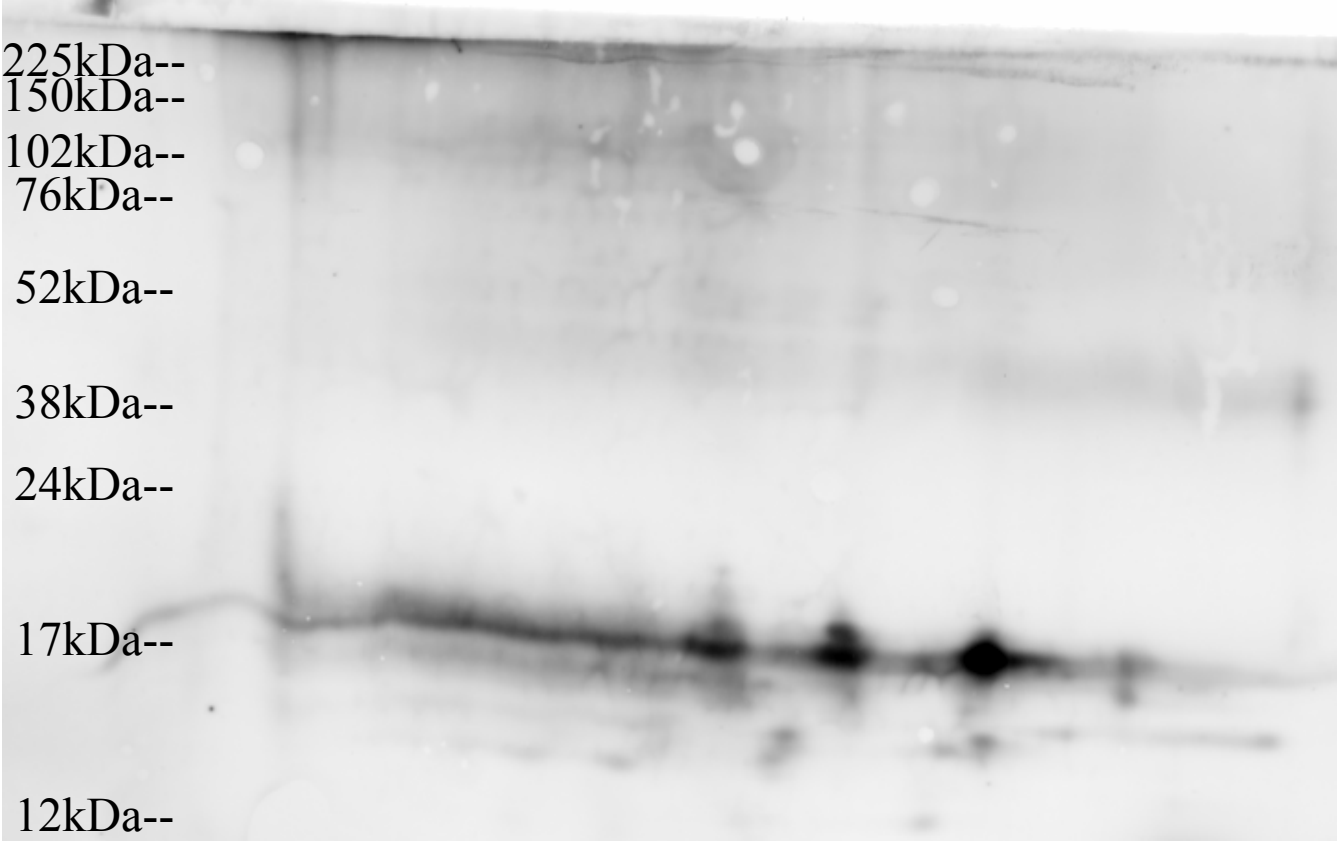

Supplement: Additional file 1: Figure S1 — Western blot for SOD1 on 2D separated spinal cord. Spinal cords from WT TG mice were homogenized and unlabeled proteins were separated by 2D gel electrophoresis according to Methods. 2D gels were then equilibrated and blotted for human SOD1. SOD1 immunoreactive spots were matched with 2D gel spots for SOD1. [file 1750-1326-8-43-S1.pdf]

Supplementary Figure 2

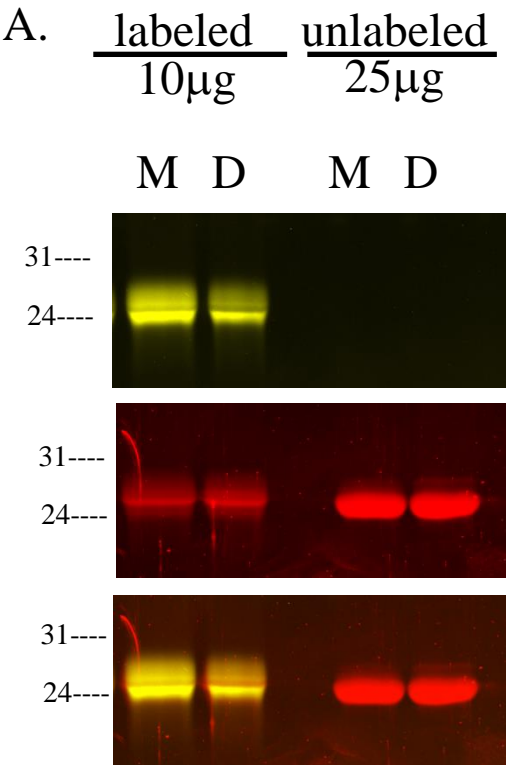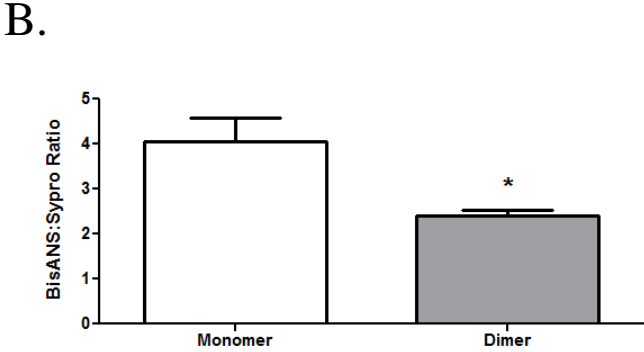

Supplement: Additional file 2: Figure S2 — Surface hydrophobicity of monomeric and dimeric UCHL1. A) Recombinant monomeric (M) and dimeric (D) UCHL1 were fractionated by size exclusion chromatography and labeled with 5-molar excess bisANS and resolved by 12% SDS page, with unlabeled UCHL1 as a control. B) The resultant bisANS fluorescence in monomeric and dimeric preparations of UCHL1 were quantitated and normalized by Sypro Ruby staining. *p<0.05 by Student’s t test. [file 1750-1326-8-43-S2.pdf]

Supplementary Figure 3

A.

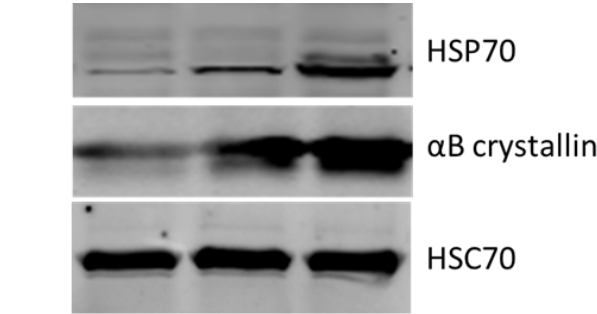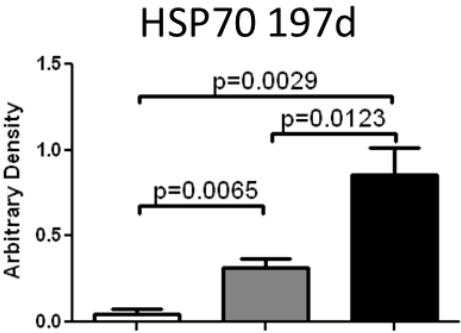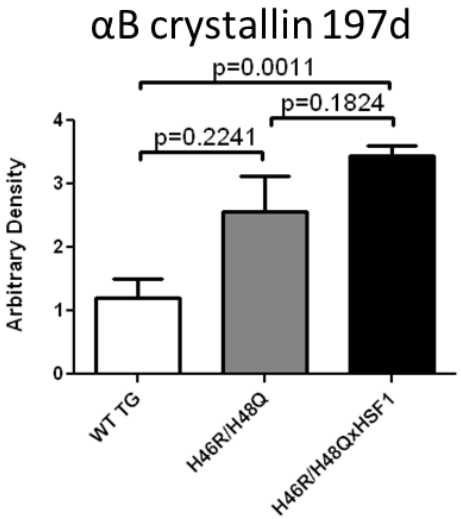

B.

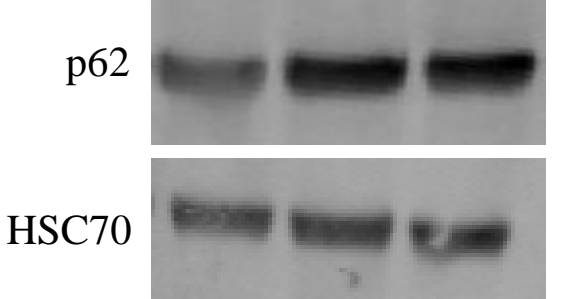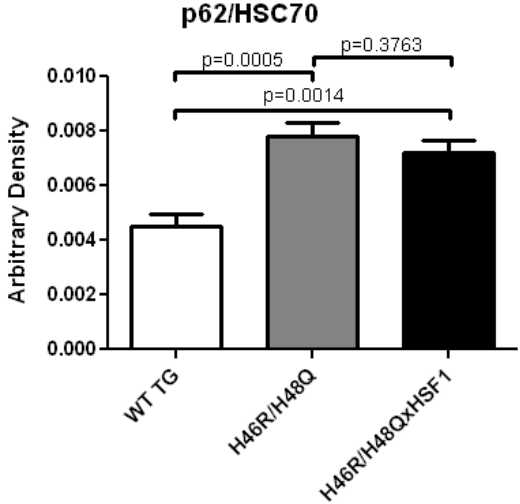

Supplement: Additional file 3: Figure S3 — Recombinant UCHL1 UCHL1 protein was prepared essentially as described [80] with the following modifications: the induced cell pellet was resuspended in 4 mL PBS buffer/L culture prior to chromatography. Cells were disrupted by passage twice through a French pressure cell (Thermo Fisher) at 12,000 psi. Two peaks containing UCHL1 eluted from the final S200 chromatography step; the earlier peak was shown to correspond to the dimer by analytical gel filtration and native PAGE, whereas the latter peak was consistent with the monomer. [file 1750-1326-8-43-S3.pdf]

Supplementary Figure 4

HSP70

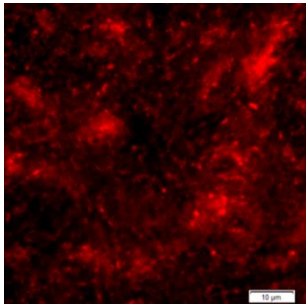

GFAP

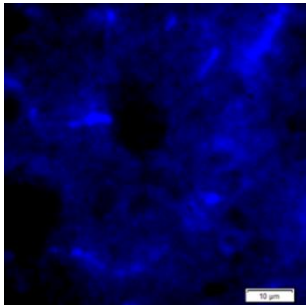

Merge

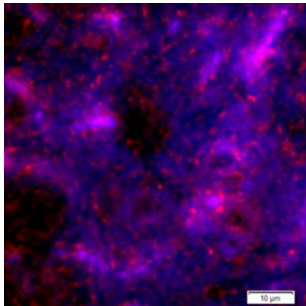

$\alpha$ B-Crystallin

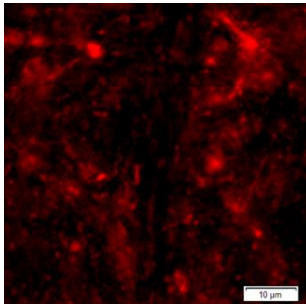

GFAP

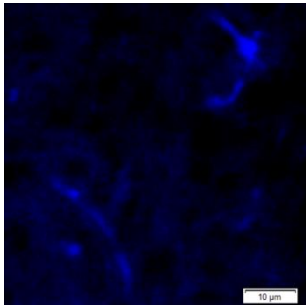

Merge

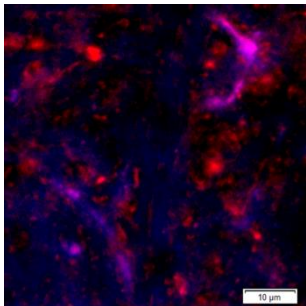

Supplement: Additional file 4: Figure S4 — Effect of HSF1 overexpression on Chaperone and p62 levels in H46R/H48Q mice. Whole spinal cords were homogenized in 2%SDS and immunoblotted for the A) HSP70 and αB-crystallin or B) p62 and normalized with Hsc70. Bars represent an n=6 +/- SD. [file 1750-1326-8-43-S4.pdf]
