# Supplementary material for: Heat shock factor 1 over-expression protects against exposure of hydrophobic residues on mutant SOD1 and early mortality in a mouse model of amyotrophic lateral sclerosis
Source: Mol Neurodegener. 2013 Nov 21;8:43. doi: 10.1186/1750-1326-8-43 (PMC3907013; doi:10.1186/1750-1326-8-43)
Supplement: Additional file 5: Figure S5 — Double immunofluorescence labeling of anterior horn lumbar spinal cord. H46R/H48QxHSF1 tissues were stained with HSP70 (red) and astrocyte marker Glial Fibrillary Acidic Protein (GFAP, blue). Some of the tissue staining for HSP70 and αB-crystallin can be accounted for by astrocytes as shown by colocalization with GFAP. Scale bar represents 10 μm. [file 1750-1326-8-43-S5.doc]

**Additional file 5**

**Recombinant UCHL1** UCHL1 protein was prepared essentially as described [82] with the following modifications: the induced cell pellet was resuspended in 4 mL PBS buffer/L culture prior to chromatography. Cells were disrupted by passage twice through a French pressure cell (Thermo Fisher) at 12,000 psi. Two peaks containing UCHL1 eluted from the final S200 chromatography step; the earlier peak was shown to correspond to the dimer by analytical gel filtration and native PAGE, whereas the latter peak was consistent with the monomer.
